# Supplementary material for: Willingness to trust is reduced by loneliness and paranoia
Source: Commun Psychol. 2025 Dec 30;4:17. doi: 10.1038/s44271-025-00384-6 (PMC12855875; doi:10.1038/s44271-025-00384-6)
Supplement: Supplementary file 2 — Supplemental Materials [file 44271_2025_384_MOESM2_ESM.pdf]

Model	Model details
1: $\tau_{2\omega}$	$u_t(i_t,\mathcal{D}_{t-1}) = e_t - (1-\tau)s_t + \tau\left(\eta_t \int_0^1 p_t(r_t \mid i_t,\mathcal{D}_{t-1})dr\right)$<br>$p_t(r_t \mid i_t,\mathcal{D}_{t-1}) = r^{\alpha_{t-1}+\omega_1 r_t-1}(1-r)^{\beta_{t-1}+\omega_2(1-r_t)-1}$<br>$p_t(i_t \mid \mathcal{D}_{t-1}) \propto e^{\chi\, u_t(i_t,\mathcal{D}_{t-1})}$
2: $\tau_{p,n}$	$\begin{cases} u_t(i_t,\mathcal{D}_{t-1}) = e_t - (1-\tau_p)s_t + \tau_p\left(\eta_t \int_0^1 p_t(r_t \mid i_t,\mathcal{D}_{t-1})dr\right), & if\ r_{t-1} \geq r_{t-2} \\ u_t(i_t,\mathcal{D}_{t-1}) = e_t - (1-\tau_n)s_t + \tau_n\left(\eta_t \int_0^1 p_t(r_t \mid i_t,\mathcal{D}_{t-1})dr\right), & if\ r_{t-1} < r_{t-2} \end{cases}$<br>$p_t(r_t \mid i_t,\mathcal{D}_{t-1}) = r^{\alpha_{t-1}+r_t-1}(1-r)^{\beta_{t-1}+(1-r_t)-1}$<br>$p_t(i_t \mid \mathcal{D}_{t-1}) \propto e^{\chi\, u_t(i_t,\mathcal{D}_{t-1})}$
3: IA	$u_t^n(i_t,r_t) = x_t^n - \zeta \max\{x_t^n - x_t^m, 0\} - \xi \max\{x_t^m - x_t^n, 0\}$<br>$p_t(i_t \mid r_t) \propto e^{\chi\, u_t^n(i_t,r_t)}$
4: Bayesian IA	$x_t^m = e_t - s_t + \eta_t \int_0^1 p_t(r_t \mid i_t,\mathcal{D}_{t-1})dr$<br>$x_t^n = \eta_t - \eta_t \int_0^1 p_t(r_t \mid i_t,\mathcal{D}_{t-1})dr$<br>$u_t^n(i_t,r_t) = x_t^n - \zeta \max\{x_t^n - x_t^m, 0\} - \xi \max\{x_t^m - x_t^n, 0\}$<br>$p_t(r_t \mid i_t,\mathcal{D}_{t-1}) = r^{\alpha_{t-1}+r_t-1}(1-r)^{\beta_{t-1}+(1-r_t)-1}$<br>$p_t(i_t \mid r_t) \propto e^{\chi\, u_t^n(i_t,r_t)}$
5: Bayesian $IA_{2\omega}$	$x_t^m = e_t - s_t + \eta_t \int_0^1 p_t(r_t \mid i_t,\mathcal{D}_{t-1})dr$<br>$x_t^n = \eta_t - \eta_t \int_0^1 p_t(r_t \mid i_t,\mathcal{D}_{t-1})dr$<br>$u_t^n(i_t,r_t) = x_t^n - \zeta \max\{x_t^n - x_t^m, 0\} - \xi \max\{x_t^m - x_t^n, 0\}$<br>$p_t(r_t \mid i_t,\mathcal{D}_{t-1}) = r^{\alpha_{t-1}+\omega_1 r_t-1}(1-r)^{\beta_{t-1}+\omega_2(1-r_t)-1}$<br>$p_t(i_t \mid r_t) \propto e^{\chi\, u_t^n(i_t,r_t)}$
6: Expectation-only $\gamma_{p,n}$	$u_t(i_t,\mathcal{D}_{t-1}) = e_t - s_t + \eta_t \int_0^1 p_t(r_t \mid i_t,\mathcal{D}_{t-1})dr$<br>$p_t(r_t \mid i_t,\mathcal{D}_{t-1}) = r^{\gamma_p(\alpha_{t-1}+r_t-1)}(1-r)^{\gamma_n(\beta_{t-1}+(1-r_t)-1)}$<br>$p_t(i_t \mid \mathcal{D}_{t-1}) \propto e^{\chi\, u_t(i_t,\mathcal{D}_{t-1})}$
7: $\tau_{p,n}$ with $\gamma_{p,n}$	$\begin{cases} u_t(i_t,\mathcal{D}_{t-1}) = e_t - (1-\tau_p)s_t + \tau_p\left(\eta_t \int_0^1 p_t(r_t \mid i_t,\mathcal{D}_{t-1})dr\right), & if\ r_{t-1} \geq r_{t-2} \\ u_t(i_t,\mathcal{D}_{t-1}) = e_t - (1-\tau_n)s_t + \tau_n\left(\eta_t \int_0^1 p_t(r_t \mid i_t,\mathcal{D}_{t-1})dr\right), & if\ r_{t-1} < r_{t-2} \end{cases}$<br>$p_t(r_t \mid i_t,\mathcal{D}_{t-1}) = r^{\gamma_p(\alpha_{t-1}+r_t-1)}(1-r)^{\gamma_n(\beta_{t-1}+(1-r_t)-1)}$<br>$p_t(i_t \mid \mathcal{D}_{t-1}) \propto e^{\chi\, u_t(i_t,\mathcal{D}_{t-1})}$

Tab. S1. Model description. List of models. Variables: u_t is the participant's utility at time t, i_t is the investment ratio at time t, $\mathcal{D}_{t-1}$ is the history of observation up to trial $t-1$, e_t is the participant's endowment, s_t is the sum sent that the participant stands to lose, η_t is the partner's endowment (the tripled amount of what shared by the participant), r_t is the partner's reciprocity ratio, ω is a subject-specific parameter to capture biased updating patterns, τ is a subject-specific parameter to capture a participant's willingness to trust, χ is a subject-specific parameter to capture a participant's choice stochasticity, x_t^n is the participant's payoff, x_t^m is the partner's payoff, ζ is subject-specific parameters quantifying a participant's advantageous

inequality (guilt), ξ is subject-specific parameters quantifying a participant's disadvantageous inequality (envy) and γ is a subject-specific decay parameter. IA: inequity aversion.

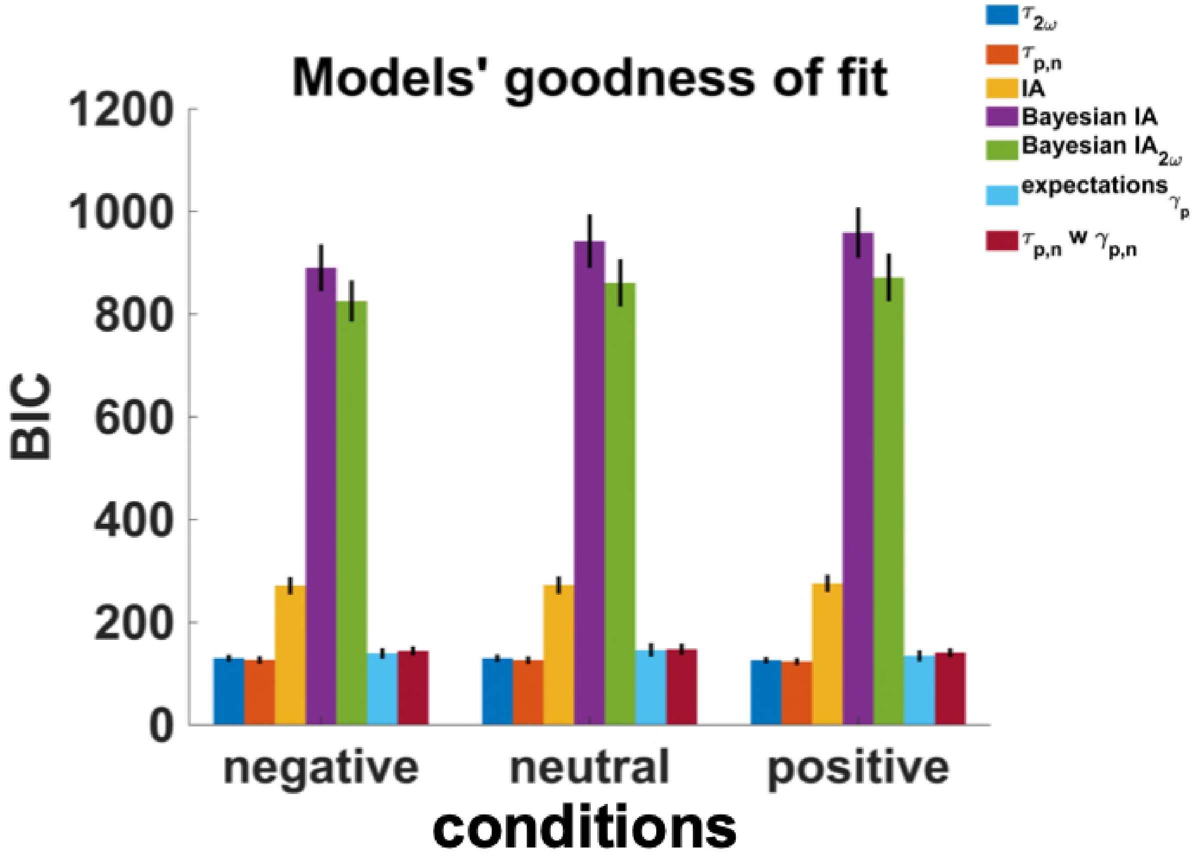

Fig. S1. Bayesian Information Criterion. Model comparison revealed that a model with two τ parameters ($\tau_{p,n}$) constituted the best tradeoff between model complexity and model fit across experimental conditions. IA: inequity aversion model

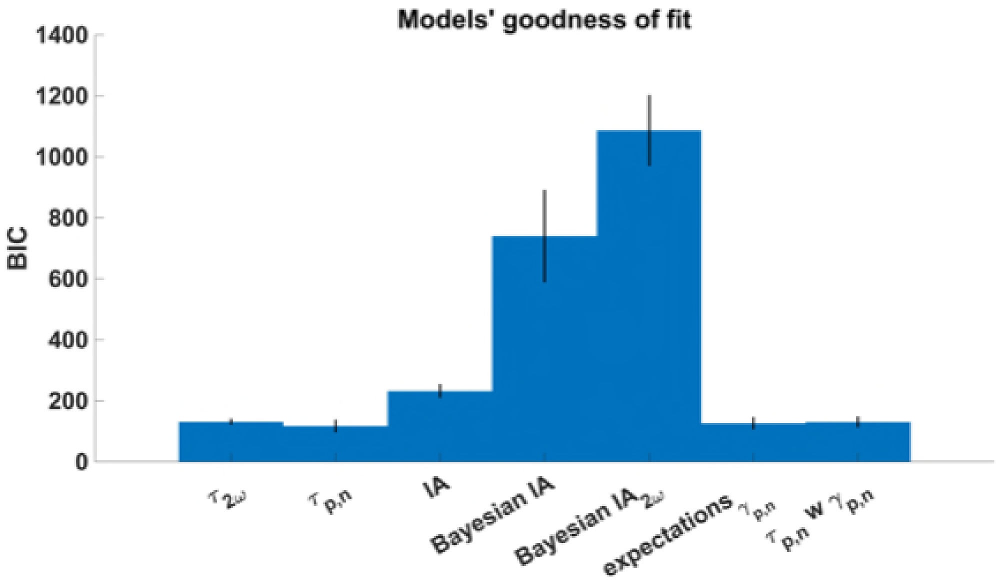

Fig. S2. Empirical values for Bayesian Information Criterion. Simulations revealed that the Bayesian Information Criterion is a well-calibrated measure to identify the winning model.

**Model** / **Condition**	$\tau_{2\omega}$	$\tau_{p,n}$	IA	Bayesian IA	Bayesian $IA_{2\omega}$	Expectation $\gamma_{p,n}$	$\tau_{p,n}$ w $\gamma_{p,n}$
Negative	130.02	126.52	270.98	890.36	825.66	139.27	144.46
Neutral	129.96	126.03	272.20	955.30	872.93	148.09	149.71
Positive	125.94	123.65	275.69	972.25	883.50	136.64	142.73

Tab. S2. Bayesian Information Criterion (BIC) Values. BIC values from the fitting procedure for the seven models tested for each of the experimental conditions
